# Study protocol of a randomized controlled trial of home-based computerized executive function training for children with cerebral palsy

**DOI:** 10.1186/s12887-019-1904-x

**Published:** 2020-01-07

**Authors:** María García-Galant, Montse Blasco, Lee Reid, Kerstin Pannek, David Leiva, Olga Laporta-Hoyos, Júlia Ballester-Plané, Júlia Miralbell, Xavi Caldú, Xènia Alonso, Esther Toro-Tamargo, Mar Meléndez-Plumed, Francisca Gimeno, Marc Coronas, Emili Soro-Camats, Roslyn Boyd, Roser Pueyo

**Affiliations:** 1 Departament de Psicologia Clínica i Psicobiologia, Universitat de Barcelona, Passeig Vall d’Hebron 171, Barcelona, 08035 Spain; 2 Institut de Neurociències, Universitat de Barcelona, Passeig Vall d’Hebron 171, Barcelona, 08035 Spain; 3 Institut de Recerca Sant Joan de Déu, Passeig de Sant Joan de Déu 2, Barcelona, 08950 Spain; 4 Australian e-Health Research Centre, Commonwealth Scientific and Industrial Research Organisation, Brockway 65, Brisbane, 6014 Queensland Australia; 5 Queensland Cerebral Palsy and Rehabilitation Research Centre, The University of Queensland, Graham 62, Brisbane, 4101 Queensland Australia; 6 Departament de Psicologia Social i Psicologia Quantitativa, Universitat de Barcelona, Barcelona, 08035 Spain; 7 Servei de Neurologia, Hospital Universitari Sant Joan de Déu, Passeig Sant Joan de Déu 2, Barcelona, 08950 Spain; 8 Servei de Rehabilitació i Medicina Física, Hospital Universitari Vall d’Hebron, Passeig Vall d’Hebron 119-129, Barcelona, 08035 Spain; 9 Serveis de Rehabilitació, Associació de la Paràlisi Cerebral (ASPACE), Camí Tres Pins 31-35, Barcelona, 08038 Spain; 10 Departament de Cognició, Desenvolupament i Psicologia de l’Educació, Universitat de Barcelona, Passeig Vall d’Hebron 171, Barcelona, 08035 Spain; 11 Unitat de Tècniques Augmentatives de Comunicació (UTAC), Universitat de Barcelona, Passeig Vall d’Hebron 171, Barcelona, 08035 Spain

**Keywords:** Cerebral palsy, Executive functions, Cognitive training, Computerized therapy, Quality of life, Neuroimaging, Participation

## Abstract

**Background:**

Cerebral palsy (CP) is frequently associated with specific cognitive impairments, such as executive dysfunction which are related to participation and quality of life (QOL). The proposed study will examine whether a computerized executive function (EF) training programme could provide superior benefits for executive functioning, participation, QOL and brain plasticity, as compared to usual care.

**Methods:**

A single-blind randomized controlled trial (RCT) design will be performed. Thirty children with CP aged 8 to 12 years will participate in a home-based computerized multi-modal executive training programme (12 weeks, 5 days a week, 30 min a day training, total dose = 30 h). Thirty children with CP matched by age, sex, motor and intelligence quotient (IQ) will compose the waitlist group. Cognitive, behavioural, emotional, participation and QOL measures will be obtained at three time points: before, immediately after and 9 months after completing the training. Additionally, structural and functional (resting state) magnetic resonance images (MRI) will be obtained in a subsample of 15 children from each group. Outcomes between groups will be compared following standard principles for RCTs.

**Discussion:**

The study will test whether the cognitive training programme exerts a positive effect not only on neuropsychological and daily functioning of children with CP but also on other measures such as participation and QOL. We will also use brain MRI to test brain functional and structural changes after the intervention.

If this on-line and home-based training programme proves effective, it could be a cost-effective intervention with short- and long-term effects on EF, participation or QOL in CP.

**Trial registration:**

ClinicalTrials.gov: NCT04025749. Registered 19 July 2019. Retrospectively registered.

## Background

Cerebral palsy (CP) describes a group of permanent disorders of the development of movement and posture, causing activity limitations that are attributed to non-progressive disturbances that occurred in the developing fetal or infant brain [1]. At present, CP is the leading cause of physical disability in children, with a prevalence of two or three out of every thousand newborns [2]. As a chronic condition, individuals with CP usually require lifetime medical, psychological, educational and social support [3]. People with CP present with alterations in sensation, perception, cognition, communication and behaviour that hinder activities of daily life, participation and quality of life (QOL) [1, 4, 5].

Cognitive functioning is the result of a complex interplay between neurological, motor and communication processes as well as environmental support over time [6]. The neuropsychological profile in CP is heterogeneous with a high prevalence of visuospatial [7] and executive function (EF) deficits [6]. Regarding EF, some studies have highlighted lower performance in sustained attention, working memory, inhibition, processing speed, metacognition and strategic planning [5, 6, 8, 9].

Executive functions play an important role in behaviour regulation, social abilities and performance of activities of daily living. In this way, poor executive functioning may lead to slower social development and behavioural problems. Executive functioning is also essential for social problem solving and emotional regulation in children and young people with CP [3, 9]. EF has been shown to play an important role in the QOL of people with CP [10].

### Home-based multi-modal computerized training in CP

Research in CP to date has mainly focused on medical- and movement-related interventions [11]. Cognitive interventions are however also needed given that cognitive impairment such as executive dysfunctions is usually associated with CP and may be related to participation and QOL.

Most current interventions require the participant to travel to receive their training, which may reduce adherence and efficacy, especially in people with motor disorders. Alternative EF home-based therapies, such as internet-delivered programmes or active videogames, are emerging as a popular modality to increase participants’ motivation [12]. Some authors suggest that home-based computerized cognitive training has the potential to deliver novel, engaging and intensive therapies to children that could improve EF performance and participation in more complex activities and their QOL [3, 4]. Computerized EF training has been demonstrated to improve EF domains such as inhibition, working memory and visuoperceptual processing in children born preterm, or with medical conditions such as Attention deficit hyperactivity disorder (ADHD), arterial ischaemic stroke, cranioencephalic trauma or cancer [13–17].

Similar results have been found in adults after computerized therapies [18–22].

According to Diamond there are three core EFs (inhibition, working memory and cognitive flexibility) and from these, higher-order EFs are built (reasoning, problem solving and planning & monitoring) [23]. There have been some home-based randomized controlled trials (RCT) in children with CP focused on a single domain of EF such as working memory using the Cogmed System [24] and attention using the MiYoga Programme [25]. Previous studies in other populations indicate that training more than one EF domain allows the transference of improvements to other cognitive functions or clinical symptoms [26, 27]. For this reason, multi-modal tasks might better allow the transference of abilities across EF domains; EF interventions should consider all components and their developmental trajectories [27].

The transference effect from motor to cognitive functions has previously been tested in the CP population. Piovesana [28] used a home-based multi-modal computerized training programme designed to improve motor skills in children with CP. They were however unable to demonstrate improvements in EF performance. Some studies that used interactive training to improve motor skills in participants with CP found changes in visuoperceptual functions [29, 30]. There is a lack of studies with long-term follow-up in this field: to our knowledge, only one study [29] has measured long-term changes in cognition (visuoperceptual function) in children with CP.

### Magnetic resonance imaging (MRI) related to cognitive function training

Measuring the changes in neuroplasticity promoted by therapy would involve understanding how and for whom rehabilitation could be effective [31]. Connectivity techniques involving diffusion MRI (dMRI) and the resting state in functional MRI (fMRI) [31, 32] have been used after motor-focused interventions in people with unilateral CP to analyse and quantify improvements. New techniques in neuroimaging might allow an understanding of the neural reorganization that occurs after a neuropsychological intervention [33].

Horowitz-Kraus [34] found that children with lector deficit increased brain activation and compensatory brain reorganization between visual and EF networks after improvements in reading skills promoted by/following computerized training. Changes in cerebral connectivity in the frontoparietal and occipital cortex networks, as well as neural connectivity changes, have also been found after cognitive training in healthy children [35–37]. Finally, Conklin [13] found changes in cerebral functioning with a reduction in the activation of the prefrontal medium and lateral cortex in children with cancer after training. In adults, some studies have found brain structural and functional changes in healthy participants after an intervention. Specifically, Eggenberger [22] induced modulations in prefrontal cortex oxygenation that were associated with improvements in EF. Another study found that exercise dose was associated with increased brain-derived neurotrophic factor as well as increased grey matter volume in the prefrontal and anterior cingulate cortices [38]. This study also found that memory improvements were associated with an increase in the volume of the dorsolateral prefrontal cortex.

## Methods

This aim of the proposed study is to conduct a single-blind RCT with 60 children with CP to assess whether a home-based computerized multi-modal training programme might be effective at improving infants’ EF, as the primary outcome. As secondary outcomes, the study will test whether the computerized therapy exerts a positive effect on other cognitive functions and other areas such as participation and QOL in children with CP. It is further expected that structural and functional brain changes might be observed.

The primary hypothesis to be tested is:

- The computerized multi-modal cognitive training programme will be more effective than usual care alone at improving executive functioning in children with CP.

The secondary hypotheses are that the computerized multi-modal cognitive training programme will be more effective than usual care at improving the following outcomes in children with CP.

The computerized multi-modal cognitive training programme will be more effective than usual care at improving the following outcomes in children with CP:

- Visuoperception and memory due to the transference effect.
- Participation.
- QOL.
- Structural and functional brain changes due to brain plasticity.

The long-term efficacy of the intervention will be checked. Finally, the effect of other variables such as the main sociodemographic and clinical factors on the efficacy of EF training will be evaluated.

### Participants

The sample will consist of 60 children with CP. These participants will mainly be recruited from the Hospital Vall d’Hebron, the Hospital Sant Joan de Déu and the Cerebral Palsy Association ASPACE in Barcelona.

#### Inclusion criteria

- Aged 8–12 years.
- Manual Abilities Classification System (MACS) ranging from I to III [39].
- Intelligible yes/no response system.
- Ability to understand simple instructions as evaluated using the Screening Test of Spanish Grammar [40].
- Availability to participate in the investigation for 1 year.
- Accessibility to the internet at home.

Participants who have no metallic prostheses, brain shunts, claustrophobia or other factors that prevent the application of neuroimaging will be prioritized to undergo scanning.

#### Exclusion criteria

- Identified hearing or visual impairment that precludes neuropsychological assessment and cognitive training.

### Procedure

Participants will be contacted by the medical staff of their reference health centre. Then, researchers will provide participant information and seek informed consent at a first interview at their reference health centre or in the study setting at the Faculty of Psychology, University of Barcelona (UB).

Assessments will be carried out at the University of Barcelona and neuroimaging will be performed at the Hospital Sant Joan de Déu. The intervention will take place in each participant’s home, with monitoring and support from an experienced neuropsychologist.

### Design

**Fig. 1:**
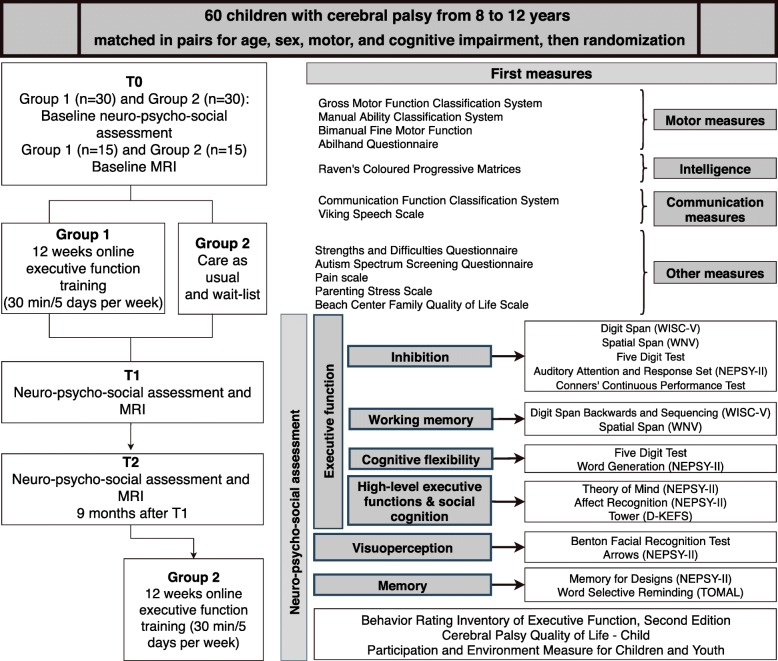
D-KEFS: Delis-Kaplan Executive Function System; MRI: Magnetic Resonance Imaging; NEPSY-II: A Developmental NEuroPSYchological Assessment-II; TOMAL: Test of Memory and Learning; WISC-V: Wechsler Intelligence Scale for Children, Fifth Edition; WNV: Wechsler Nonverbal Scale of Ability

This study is researcher-blinded and will be a pair-matched and randomized waitlist controlled trial. Demographic data and motor, communication and other associated deficit measures will be collected at the first interview after participants’ parents or guardians and children have provided informed consent. Detailed information about the methodology and timing of assessments is shown in Fig. 1. The waitlist design will ensure that all participants have access to this novel intervention by assigning each participant to either Group 1 (immediate intervention) or Group 2 (waitlist */* delayed intervention)*.*

After baseline assessments (T0, Additional file 1), the Group 2 will continue care as usual for 12 weeks, whilst the Group 1 will receive usual care as well as home-based multi- modal cognitive training for 12 weeks (30 min/day, 5 days/week, total of 30 h). Both groups will be assessed after these 12 weeks (T1). A follow-up assessment will be performed 9 months after T1 (T2). After this assessment, Group 2 will have access to the same conditions as Group 1.

Patients or the public were not involved in the design, or conduct, or reporting, or dissemination of our research.

### Sample size determination

The required sample size was calculated by taking into account the differences in the continuous primary outcomes after 12 weeks of following the computerized training programme. Simulations under two different scenarios were carried out. Specifically, calculations for separate tests and multiple-end-points were performed. By comparing the two scenarios, conservative figures regarding sample size were estimated. As a result, to detect a large standardized difference (i.e. a difference of at least 0.8 SD) between the immediate intervention and waitlist groups, with 80% power and α = 0.05, 26 children would be necessary in each group. Assuming 15% attrition, the required sample size was calculated as 60 participants.

### Randomization

Participants will be matched in pairs according to age, sex, IQ and manual ability. Each member of the pair will then be randomly allocated to one of the two groups. Randomization will be carried out using an in-house program written in R, DL will generate the allocation sequence and assign participants to interventions. Once the randomization process is completed, MG-G will inform the participants’ parents and guardians of their group allocation as well as provide the details of the computerized cognitive training programme, if applicable, to their families.

### Blinding

Single blinding will be applied to the researcher that performs the cognitive assessment as well as those in charge of the statistical analyses. Thus, code identification will be used for the two groups, ensuring that correspondence between codes and group characteristics (i.e. immediate intervention or waitlist) is not known by the aforementioned individuals.

### Adverse events

Any minor or major events associated with the intervention or usual care groups will be screened every week through open-ended questions. Any adverse events or unintended effects detected will be reviewed by a researcher.

### Equipment

The cognitive training programme requires no specialized resources other than an internet connection. All participants will receive a password to access the home-based computerized multi-modal training programme.

### Computerized multi-modal cognitive training

Neuron Up (www.neuronup.com) is the cognitive training and stimulation programme that will be used. It covers all levels of difficulty of all EF domains. Table 1 shows some examples of the tasks. The training programme will also include some cognitive tasks based on activities of daily living that may promote generalization. The programme is delivered via the internet, providing the possibility of monitoring the participants’ performance in real time.

**Table 1:**
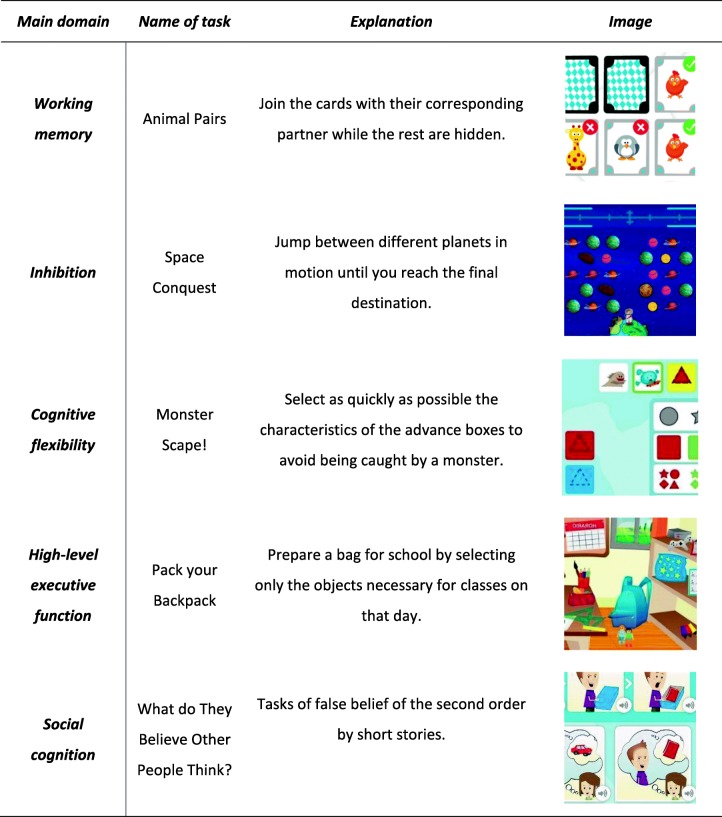
Examples of multi-modal activities in home-based computerized cognitive training programme

A participants’ instruction manual explaining how the training programme works, and providing contact information and motivational strategies to encourage engagement in the cognitive training programme, will be delivered to each participant. A trainer will be able to monitor each participant’s training, and record important data such as how frequently the participant has logged into NeuronUp, how long they have spent engaged in the therapy, their training progress and their level of motivation. Neuropsychologist will contact each participant’s family weekly in order to give feedback on the recorded information.

### Data management

All collected data will be managed through a confidential online database run through the Universitat de Barcelona. Files containing information from the participants will be stored in a locked filing cabinet at the Universitat de Barcelona. Each participant will use a personal ID to log into NeuronUp, and the study researcher who will contact the families and follow up the training (neuropsychologist) will be different from the researcher that performs the cognitive assessment.

### Assessments

Figure 1 shows the neuro-psycho-social assessments that will be performed at T0, T1 and T2. Effort has been made to select cognitive measures that are free of motor and speed components. Several domains of EF, as well as visuoperception, memory, participation and QOL will be assessed. The measures used are standardized and validated for use across a wide age range and have good retest reliability (Additional file 1).

As Sabbadini [41] advised, participants will be encouraged to answer by themselves, but some accommodations will be used if necessary, taking into account the experience of previous studies [42].

### MRI acquisition

Neuroimaging will be performed at T0, T1 and T2. Participants will be prepared for the imaging sessions in order to optimize the quality of images acquired. This preparatory session will consist of explaining the procedure, familiarization with the facilities and finding entertainment strategies adapted to each participant.

Structural, functional, and diffusion MR images will be acquired on a Phillips Ingenia 3.0 T scanner. These sequences have been harmonised with several other studies [43–46] in order to maximise their utility and interpretability [47].

Structural imaging includes high resolution T1w MPRAGE (FOV 256 mm; acquisition matrix size 256 × 255; reconstruction matrix size 256 × 256; 192 slices; slice thickness 1 mm; TR 2500 ms; TE 3.0 ms; flip angle 9°; SENSE acceleration factor: RL 2) and high resolution T2w FLAIR (FOV 256 mm; acquisition / reconstruction matrices sized 256 × 256; 192 slices; slice thickness 1 mm; TR 5000 ms; TE 388 ms; SENSE acceleration factors: AP 1.4, RL 2).

The diffusion weighted imaging protocol is a multishell sequence (FOV 240 mm; acquisition matrix size 96 × 94; reconstruction matrix size 96 × 96; 60 slices; slice thickness 2.5 mm; TR 8778 ms; TE 115 ms; flip angle 90°; SENSE acceleration factor: AP 2). The acquisition includes 8x non-diffusion weighted images (b = 0 s/mm^2^) as well as 20 (b = 1000s/mm^2^) and 60 (b = 3000 s/mm^2^) unique directions. These are split across four blocks with alternating AP/PA phase encoding directions for the purpose of post-acquisition distortion correction. Acquiring images across four blocks also allows technicians to re-acquire portions of sequence if the child moves during acquisition, rather than requiring re-acquisition of the full sequence.

A resting state fMRI will also be acquired (FOV 240 mm; acquisition matrix size 80 × 78; reconstruction matrix size 80 × 80; 40 slices; slice thickness 3 mm; TR 2300 ms; TE 30 ms; SENSE acceleration factor, AP 2; 266 frames). Participants will be asked to remain still and relaxed with their eyes closed for the total duration of this scan.

### Statistical analysis

Statistical analysis will follow standard principles for RCTs (e.g. running sensitivity analysis with non-ignorable missing data). Primary and secondary outcomes will be summarized for each group depending on the measurement scale and data distribution (frequencies, means, SDs, medians, IQRs, 95% CIs). To assess training effectiveness, parametric (or nonparametric, depending on the data distribution) tests will be used for the groupwise comparisons after the 12th week (post-intervention; T1). Comparisons between groups at follow-up (after 9 months; T2) will be made using Generalized Estimating Equations (GEEs). The fixed factors are time (week 0, 12, and 36), group (immediate and waitlist), and time by group interaction. Assuming a Gaussian distribution for the outcomes, identity link functions will be used. Given the small sample size and in order to avoid possible numerical problems, we will reduce the complexity of the model by using an exchangeable working correlation matrix. Statistical significance will be considered at *p* < .05 and post-hoc adjustment will be applied for multiple comparisons. All data will be treated following an intention-to-treat approach and multiple imputation will be used for treating missing data whenever possible.

Finally we will compare neuroimaging data achieved from structural sequences as T1w, T2w FLAIR and dMRI and functional acquisitions between the groups and with each other. For this purpose appropriate preprocessing and adequate analysis of data will also be performed.

## Discussion

This protocol describes a RCT to test whether cognitive training (30 h/12 weeks) exerts a positive effect not only on the cognitive and daily functioning of children with CP but also on other measures such as participation and QOL. We will further test whether there are changes in brain functions and structure following cognitive training.

This protocol has four key strengths. Firstly, it will be the first study of CP that analyses the functional and structural brain changes due to plasticity after performing cognitive training.

Secondly, it will be a single-blind RCT to avoid biases. Thirdly, the therapy will be home-based computerized multi-modal cognitive training, which provides the opportunity to increase the length of sessions and, at the same time, maximize training adherence [12]. Finally, this will be one of only a few studies so far published in CP to analyse the long-term results after training, providing information on the long-term neuropsychological effects and the maintenance of their benefits on daily functioning of children with CP but also on participation and QOL.

This protocol also has some limitations. First, the anticipated small sample size may be too small to detect brain changes. Consequently, to improve statistical power we will carefully consider the preparation of participants for scanning, and the image processing methods [31]. A further limitation is the lack of an active control group. This is due to the difficulty in finding or developing an alternative cognitive task completely free of EF training. Instead, this study will compare the effect of care as usual to the effect of adding a home-based computerized multi-modal cognitive training programme to determine what this additional therapy adds, if anything.

In conclusion, if this on-line and home-based training proves to be effective, it could be a cost-effective intervention for children with CP and their families, with near effects on EF and far effects on memory and visuospatial functions and on participation or QOL that are easily accessible for all.

## Supplementary information

**Additional file 1.** Motor, communication, intelligence and other measures at T0 assessments. Table with name and brief description of each assessment tool in T0. Neuropsychological tests and parental questionnaires at T0, T1 and T2 assessments. Table with name, brief description and reliability information of each assessment tool at T0, T1 and T2.

## Data Availability

Not applicable, as this is a protocol manuscript.

## Abbreviations

CP: Cerebral Palsy
EF: Executive Function
GEEs: Generalized Estimating Equations.
IQ: Intelligence Quotient
MACS: Manual Abilities Classification System
MRI: Magnetic Resonance Imaging
QoL: Quality Of Life
RCT: Randomized Controlled Trial
UB: University of Barcelona
