# Supplementary material for: Study protocol of a randomized controlled trial of home-based computerized executive function training for children with cerebral palsy
Source: BMC Pediatr. 2020 Jan 7;20:9. doi: 10.1186/s12887-019-1904-x (PMC6945450; doi:10.1186/s12887-019-1904-x)
Supplement: Supplementary file 1 — Additional file 1. Motor, communication, intelligence and other measures at T0 assessments. Table with name and brief description of each assessment tool in T0. Neuropsychological tests and parental questionnaires at T0, T1 and T2 assessments. Table with name, brief description and reliability information of each assessment tool at T0, T1 and T2. [file 12887_2019_1904_MOESM1_ESM.pdf]

Additional file 1. Motor, communication, intelligence and other measures at T0 assessments.

INSTRUMENT	BRIEF DESCRIPTION
MOTOR MEASURES	
Gross Motor Function Classification (GMFCS)	GMFCS is a five level classification system of children's functional gross motor severity. It is based on patient's self-initiated movement with emphasis on sitting, transfers and mobility (1).
Manual Ability Classification System (MACS)	MACS is a five level classification system based on the children's self-initiated ability to handle objects and their need for assistance or adaptation to perform manual activities in everyday life (2).
Bimanual Fine Motor Function (BFMF)	BFMF classification level is determined by assessing the child's ability to grasp, manipulate, and hold objects for each hand. Five ordinal grading levels of fine motor function are obtained (3).
Abilhand Questionnaire	It is a measure by 21 items of manual ability for children with upper limb impairments and the person's ability to manage daily activities that require the use of upper limb, whatever the strategies involved (4).
COMMUNICATION MEASURES	
Communication Function Classification System	This daily communication abilities scale ranges from I to V levels of communication in terms of effectiveness and velocity of the communication (5).
Viking Speech Scale	It is a scale to classify children's speech production. Viking Speech Scale describes four levels (6).
INTELLIGENCE	
Raven's Coloured Progressive Matrices	This test consists of 36 items, grouped into three sets of 12 items of increasing difficulty within each set. Each item contains a pattern problem with one part removed. The participant has to choose which of the six alternatives completes the pattern (7). It has been proved that this test gives comprehensive information on cognitive performance in CP, comprising not only visual but also verbal functions (8).
OTHER MEASURES	
Strengths and Difficulties Questionnaire	This questionnaire is used to assess parents' perceptions of pro-social and difficult behaviours in their child. Five sub-scales are considered: frequency of emotional symptoms; conduct problems; inattention/hyperactivity; peer problems; and prosocial behaviour (9).
Autism Spectrum Screening Questionaire	It is a screening questionnaire used to identify autism spectrum symptoms in high-functioning children and adolescents (10).
Pain Scale	It is a scale to assess the intensity of pain in children with CP. Firstly, parents were asked if the child had experienced any pain anytime and during the last month. Secondly, participants responded the two questions on pain about intensity and frequency during the last month using the Child Health Questionnaire as in the Study of Participation of Children with CP living in Europe (SPARCLE) (11).
Parental Stress Scale	It is a self-report scale that measures changes in parental stress levels for careers who have accessed targeted support, such as family support, parenting courses and one to one parenting support (12).
Beach Center Family Quality of Life Scale	This questionnaire assesses families' perceptions of their satisfaction with different aspects of family quality of life. It contains five subscales: family interaction, parenting, emotional wellbeing, physical/material wellbeing and disability-related support (13).

Additional file. Neuropsychological tests and parental questionnaires at T0, T1 and T2 assessments.

NEURO-PSYCHO-SOCIAL MEASURES		
INSTRUMENT	BRIEF DESCRIPTION	RELIABILITY
**Inhibition and Working memory**		
Spatial Span subtest (WNS)	It comprises two series: the forward condition, in which the examiner points out some cubes and the examinee must indicate the same cubes in the same order; and backward, in which the examinee must indicate the cubes in the reverse order. In both conditions, the length of the sequences is gradually increased.	The reliability of the subtest is 0.73 (14).
Digit Span subtest (WISC-V)	It comprises two series: the forward condition, in which the person who perform the assessment read a sequence of numbers and the examinee must recall the numbers in the same order; and the backward condition, in which the examinee must recall the numbers in reverse order.	The reliability of the cognitive competence index (CCI), composed of the subtest of digits, is 0.92 (15).
Conner's Continuous Performance Test - II	In this task respondents are required to press whenever any letter except the target letter 'X' appears on the computer screen.	The reliability of this test is 0.66-0.95 (16).
Auditory Attention and Response Set (NEPSY- II)	The task consists in listening a series of words and when a target word is listened the examinee must touch the correspondent circle associated.	The reliability of this subindex is 0.87 (17).
**Cognitive flexibility**		
Five Digit Test	It is considered as an alternative for the Stroop Test. FDT is a multilingual non-reading test to assess cognitive flexibility and inhibition. It minimizes the effects of education and social class and allows the testing of some severe clinical cases.	The reliability for this test is 0.86-0.94 (18).
Word Generation (NEPSY-II)	This subindex is aimed to evaluate the acquisition and recuperation of language, recuperation of the vocabulary, spelling ability, starting, working memory, speed of processing, attention, sustained effort and verbal productivity through a task based on generate words that belong to a certain semantic category or with a specific initial letter.	This subindex has a reliability of 0.79 (17).
**High-level Executive Functions**		
Tower (D-KEFS)	The Tower Test is a modification similar to earlier Tower test, that is, Tower of Hanoi. The D-KEFS version's usefulness in comparison to these other version is not documented, but D-KEFS version will prove more child-friendly that these other tower tasks remains to be seen (Sue Baron, 2004).	The reliability for the population between 8 to 19 years is 0.51; in population with CP is 0.74 (19).
**Social Cognition**		
Theory of mind (NEPSY-II)	This subtest evaluates social perception through assessing the ability to understand mental functions such as believes intention, hoax, emotion, imagination and pretending. The tasks consist in a series of questions about images in which the subject must understand the other person point of view.	The reliability of this test is 0.65-0.70 (17).
Affect recognition (NEPSY-II)	This subindex evaluates the ability of recognise emotions through pictures of kids' faces.	The reliability of this test is 0.63-0.97 (17).
**Memory**		
Memory for Designs (NEPSY-II)	This subindex evaluates the ability of learn and remember visual information of children.	The reliability of this test is 0.74-0.88 (17).
Word Selective Reminding (TOMAL)	This subindex evaluates the ability of learn and remember verbal information of children.	The reliability of this test is 0.96 (20).
**Visuospatial processing**		
Arrows (NEPSY-II)	This subindex consists in judging the orientation of a line. Several arrows are disposed around a dot and the examinee has to say which lines are addressed towards the dot.	The reliability of this test is 0.74-0.88 (17).

Benton's Facial Recognition test	Subjects are presented with a target face above six test faces, and they are asked to indicate which of the six images match the target face.	Part-whole correlation coefficients between the short and long forms of the test ranged from 0.884 to 0.940 (21).
Parental Questionnaires		
Behavior Rating Inventory of Executive Function, Second Edition	BRIEF is a parent-rated questionnaire designed to assess the behavioural manifestations of executive functions in everyday life. It consists of eight subscales which combine to form the Behavioural Regulation Index and the Metacognition and Global index.	The reliability is on the 0.90 (22).
Cerebral Palsy Quality of Life	CP QOL refers to an individual's perception of their wellbeing across various domains of life. The topics are: social wellbeing and acceptance; feelings about functioning; participation and physical health; emotional wellbeing and self-esteem; access to services; pain and impact of disability and family health (22).	The reliability for primary caregivers is 0.76 – 0.89 (23).
Participation and Environment Measure for Children and Youth	It is used to examine the frequency and level of participation in home, school and community settings (23). The measure also addresses whether the surrounding environment makes it easier or harder to participate.	This scale reported moderate to good internal consistency (0.59) and reliability (0.58) (24).

REFERENCES

1. Palisano R, Rosenbaum P, Walter S, Russell D, Wood E, Galuppi B. Gross motor function classification system for cerebral palsy. Dev Med Child Neurol. 1997;39(4): 214-23.

2. Eliasson AC, Krumlinde-Sundholm L, Rösblad B, Beckung E, Arner M, Öhrvall A-M, et al. The Manual Ability Classification System (MACS) for children with cerebral palsy: scale development and evidence of validity and reliability. Dev Med Child Neurol.2006;48(7):549–54.

3. Elvrum AKG, Andersen GL, Himmelmann K, Beckung E, Ohrvall A-M, Lydersen S, et al. Bimanual Fine Motor Function (BFMF) Classification in Children with Cerebral Palsy: Aspects of Construct and Content Validity. Phys Occup Ther Pediatr. 2016;36(1):1–16

4. Arnould C, Penta M, Renders A, Thonnard JL. ABILHAND-Kids: A measure of manual ability in children with cerebral palsy. Neurology. 2004;63(6):1045–52.

5. Vander Zwart KE, Geytenbeek JJ, de Kleijn M, Oostrom KJ, Gorter JW, Hidecker MJC, et al. Reliability of the Dutch-language version of the Communication Function Classification System and its association with language comprehension and method of communication. Dev Med Child Neurol. 2016;58(2):180–8.

6. Pennington L, Virella D, Mjøen T, da Graça Andrada M, Murray J, Colver A, et al. Development of The Viking Speech Scale to classify the speech of children with cerebral palsy. Res Dev Disabil. 2013;34(10):3202-10.

7. Raven JC, Court JH, Seisdedos Cubero N. Raven matrices progresivas escalas: CPM color, SPM general, APM superior. Madrid : TEA Ediciones; 2001.

8. Ballester-Plané J, Laporta-Hoyos O, Macaya A, Póo P, Meléndez-Plumed M, Vázquez É, et al. Measuring intellectual ability in cerebral palsy: The comparison of three tests and their neuroimaging correlates. Research in Developmental Disabilities. 2016;56:83–98.

9. Goodman R. Psychometric properties of the strengths and difficulties questionnaire. J Am Acad Child Adolesc Psychiatry. 2001;40(11):1337–45.

10. Mattila M-L, Jussila K, Linna S-L, Kielinen M, Bloigu R, Kuusikko-Gauffin S, et al. Validation of the Finnish Autism Spectrum Screening Questionnaire (ASSQ) for Clinical Settings and Total Population Screening. Journal of Autism and Developmental Disorders. 2012;42(10):2162–80.

11. Parkinson K, Gibson L, Dickinson H, Colver A. Pain in children with cerebral palsy: a cross-sectional multicentre European study. Acta Paediatrica. 2010;99(3):446–51.

12. Berry JO, Jones WH. The Parental Stress Scale: Initial Psychometric Evidence. Journal of Social and Personal Relationships.1995;12(3):463–72.

13. Hoffman L, Marquis J, Poston D, Summers JA, Turnbull A. Assessing Family Outcomes: Psychometric Evaluation of the Beach Center Family Quality of Life Scale. J Marriage Fam. 2006;68(4):1069–83.

14. Wechsler D, Naglieri JA. Wechsler Nonverbal Scale of Ability (WNV). Pearson; 2006.

15. Szczepkowski K, Demakis G. Does Executive Functioning Predict Financial Capacity in College Students Beyond Inancial Literacy, Mathematical Ability, and Estimated IQ? Archives of Clinical Neuropsychology. 2017;32(6):667–765.

16. Conners CK. Conners Continuous Performance Test II Version 5™. PsycTESTS Dataset; 2012.

17. Korkman M, Kirk U, Kemp S. NEPSY-II. Madrid: Pearson; 2014.

18. Sedó M, De Paula J, Malloy-Diniz L.F. Five digit test. Madrid: TEA; 2007.

19. Delis DC, Kaplan E, Kramer JH. Manual for the Delis-Kaplan Executive Function System (D-KEFS). San Antonio, TX: Psychological Corp;2001.

20. Reynolds & Cecil R. Reliability of Performance on the Test of Memory and Learning (TOMAL) by an Adolescent Learning Disability Sample. Educational and Psychological Measurement- 1998; 58(5): 832–835p.

21. Benton AL. Contributions to neuropsychological assessment: a clinical manual. Vol. 2. New York etc.: Oxford University Press; 1994. 159 p.

22. Gioia GA, Isquith PK, Guy SC, Kenworthy L. TEST REVIEW Behavior Rating Inventory of Executive Function. Child Neuropsychol.2000;6(3):235–8.

23. Davis E, Shelly A, Waters E, Davern M. Measuring the quality of life of children with cerebral palsy: comparing the conceptual differences and psychometric properties of three instruments. Dev Med Child Neurol. 2010 Feb;52(2):174–80.

24. Coster W, Bedell G, Law M, Khetani MA, Teplicky R, Liljenquist K, et al. Psychometric evaluation of the Participation and Environment Measure for Children and Youth. Dev Med Child Neurol. 2011;53(11):1030–7.
